# Microcomputers in a water authority laboratory

**DOI:** 10.1155/S1463924685000426

**Published:** 1985

**Authors:** M. P. Bertenshaw, K. C. Wheatstone

**Affiliations:** Severn-Trent Water Authority Lower Severn Divisional Laboratory 141 Church Street Worcestershire Malvern WR14 2AN UK


Journal of Automatic Chemistry/Journal of Clinical Laboratory Automation, Vol. 7, No. 4 (October–December 1985), pp. 192–196

### Microcomputers in a water authority laboratory

**M. P. Bertenshaw and K. C. Wheatstone**

*Severn–Trent Water Authority, Lower Severn Divisional Laboratory, 141 Church Street, Malvern, Worcestershire WR14 2AN, UK*

### Introduction

Water authorities in the UK are required not only to provide a potable water supply of high standard, but also to monitor and maintain standards of quality in all other aspects of their activities. This involves analysis of a very large number of samples from all parts of the hydrological cycle, from rivers and domestic supplies to sewage sludges and trade effluents. For example, the Severn–Trent Water Authority, one of 10 in England and Wales, carries out around 2.5 M chemical and bacteriological determinations per year. Many determinations are required under European Economic Community (EEC) legislation, particularly in connection with water supply; however, most information is used in operational investigations, works management, assessing charges for treatment of trade effluents, or environmental quality control.

Samples for analysis are submitted to water authority laboratories from where the results are transmitted to local or central computers for immediate examination by data users; these include operational scientists, water-supply and reclamation operational controllers and pollution-control inspectors. Data archives are maintained and updated so that they may be subsequently examined for trend analysis and long-term statistical reporting. In addition, Part 2 of the 1974 Control of Pollution Act (UK) requires that a publically accessible register of analytical data must be made available by all water authorities.

Many water authority laboratories currently use some form of computerized data management system for scheduling and validating analyses prior to reporting and archiving. Manual (keyboard) entry of results has, until recently, been the most common means of mounting information onto laboratory computers: this is not only extremely tedious for the operator but also contributes a major source of error to reported data. Although laboratory instruments used in routine analysis are normally fitted with a communications facility suitable for connection to a computer or printer, the laboratory computer cannot necessarily be configured to receive and collate data directly from such instruments since data produced usually requires some intermediate processing. Furthermore, dedicated interfacing of several laboratory instruments to a central laboratory computer makes inefficient use of the computer's time and resources and may even disrupt its primary data management role. The availability of inexpensive microcomputers with suitable I/O interfaces now makes it economical to dedicate a microcomputer to each instrument for data aquisition and processing prior to downloading to the central laboratory computer. High priority has therefore been given to investigating means of automatic data capture and this paper describes several areas of analysis in which microcomputers have been implemented in the Severn–Trent Water Authority's Lower Severn Divisional Laboratory at Malvern (figure 1).

The Malvern Laboratory carries out over 300 000 determinations per year, of which more than 200 000 are obtained through microprocessor facilities. Although most of the applications below illustrate clear labour-saving advantages which may be easily quantified, it is hoped that the reader will also appreciate the considerable savings in both time and convenience in improved data reliability over that experienced with manual data entry. Furthermore, it should be appreciated that 'in-lab' data processing, through intermediate microcomputers, gives the analyst greater freedom concerning choice of instruments and selection of analytical methods.

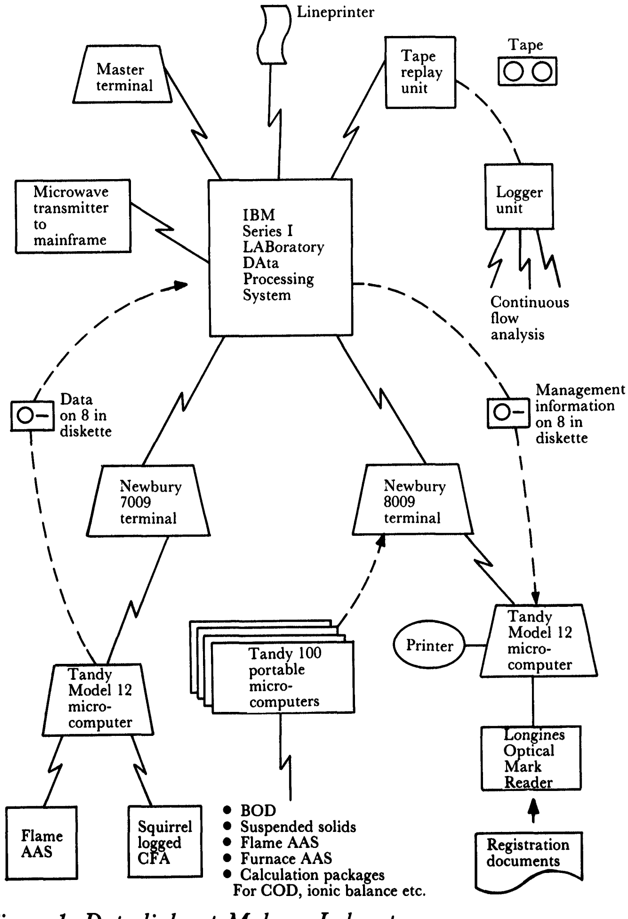

*Figure 1. Data links at Malvern Laboratory.*

### Laboratory data management

Malvern Laboratory is one of five laboratories in Severn–Trent Water Authority using LABDAPS (LABoratory DAta Processing System). This system has been developed over several years by Severn–Trent data processing staff, in collaboration with laboratory scientists. It provides most of the data management requirements of an analytical laboratory; these include registration of samples, scheduling of analyses, validation of results, production of reports, management statistics and file maintenance procedures. The software cannot be easily modified by laboratory staff since it is written in PL/I. It does, however, cater for nearly all possible individual requirements such as worksheet and report layouts, in addition to essential reference file information such as sampling site descriptions, analysis suite details and determinand reporting limits.

LABDAPS runs on an IBM Series/1 minicomputer which, at Malvern, uses 256 Kbyte core with 29 Mbyte hard disk storage configured for three full multi-tasking users. Peripherals include Newbury 7009 and 8009 terminals, a Centronics 6080 lineprinter, an IBM 8 in floppy disk drive and a Penny and Giles 2100 tape replay unit. A modem unit gives access to a microwave transmitter through which information is sent to a divisional mainframe computer; some information is also transmitted to a regional water quality archive. For automatic data capture, a rigid format must be adopted for each of the three modes of transfer permitted: keyboard, 8 in diskette, or tape replay.

Manual keyboard input of data is performed through a sequence of pre-formatted screens in which each result is validated against previously derived limits held on a file. Results are entered against laboratory worksheets; these are documents identified uniquely by number, each listing a batch of samples scheduled for a certain determination or set of determinations. It is possible to simulate keyboard entry of characters through the RS232C link intended for a printer on Newbury terminals. Serial transmission to a terminal therefore provides the simplest channel of communication from a laboratory microcomputer.

Alternatively, worksheets from the Series/1 computer can be transferred using the diskette facility by copying onto 8 in diskettes in IBM3740 format. This is a diskette exchange standard for transfer of data between different computers and is provided by most major manufacturers. The diskette is copied onto a worksheet file on the microcomputer into which results are captured. The file is then output to diskette in IBM 3740 format to be read by the Series/1 computer. Owing to this additional file-transfer step between computers, known as 'reformatting', diskette transfer is reserved for large batches of data (typically 200–600 data items).

The tape replay facility is specifically intended for use with raw data from continuous flow analysis (CFA) systems and runs in conjunction with bespoke software on the Series/1. Through this facility, the Series/1 can process up to 10 channels of CFA data simultaneously.

### Microcomputer hardware

Tandy Model 100 portable computers are used in several applications described below (see also figure 1). These mains/battery-operated microcomputers incorporate a 40 × 8 character liquid-crystal screen, an RS232C port for I/O, a parallel printer port and a cassette recorder socket. A 32K read only memory (ROM) supports Microsoft BASIC, text editing, communications and file-handling facilities. Random access memory (RAM) can be expanded from 8K to 32K and is protected by internal batteries.

Tandy Model 12 computers are used in applications where large amounts of data are to be manipulated. These incorporate an 80 × 24 character screen, two RS232C ports, a parallel printer port, dual 8 in floppy disk drives and a 64K RAM. All programs are written in Microsoft BASIC, with additional Z80 machine-code I/O routines.

Processed raw data are handled by the microcomputers in two ways: sequentially or by direct access. The Tandy Model 100 portable computer supports only sequential file access in its solid-state memory. This type of file is suitable for uploading serial data to the laboratory computer by RS232C link. However, changes to a sequential file, such as a result amendment or deletion, are complex because the entire file must be searched in sequence for the data item to be edited, then a new file created. (In practice, the original file is renamed before opening for input so that output is made to the original filename.) Also, serial transmission times can become unacceptably long since the Series/1 validates results during data entry.

For large data files, it is more convenient to use direct file access and diskette data transfer. This necessitates the use of more sophisticated hardware, for example, at Malvern Laboratory, the Model 12 computer. Editing routines are much faster using this type of data storage and more efficient data transfer can be made to the laboratory computer using the 8 in diskette facility described above.

### Sample registration

The registration of a single sample onto a laboratory data management system is by no means straightforward since this interacts with nearly all files on the computer. Consequently, simultaneous receipt of many samples from a variety of sources can become one of the most complex and time-consuming tasks in laboratory data processing. In order to achieve smooth and efficient registration of samples, analytical requirements and sample details should be recorded as quickly and accurately as possible. LABDAPS provide a facility for pre-booking known sample runs; this facility is used whenever possible and can show considerable benefits. However, most samples are received without prior knowledge or are taken on such an irregular or unpredictable basis that it is inappropriate to record them in the sample runs file. Therefore the majority of samples have to be registered individually.

A Longines Data 3000 optical mark reader (OMR), which accepts sample input documents specially designed for the purpose, is now being evaluated at the Malvern Laboratory. Sampling officers fill in these forms in the field by marking prescribed boxes in pencil. At the laboratory, the forms are fed through the OMR in which information is coded and transmitted to a Model 12 microcomputer for further processing. After automatically validating the information, the microcomputer registers samples onto LABDAPS by serial transmission of data through a sequence of pre-formatted screens, which are normally used for manual registration from a keyboard. Since information is passed from input documents to the laboratory computer with no input from the computer operator, details given on the forms by our customers are accurately recorded.

### Atomic absorption spectrophotometry

Most modern atomic absorption spectrophotometers (AAS) are fitted with internal, dedicated microprocessors. A microcomputer may be connected to the instrument for data collection and processing, normally using an RS232C link. AAS instruments interfaced to microcomputers at Malvern include Instrumentation Laboratory 157 and 457 models and a Varian 875. Output takes the same format as would be found on a printer; characters sent down the RS232C port include messages such as 'MEAN' or 'RSD', line-feeds, sample numbers and numeric results. The instruments are always configured to output the mean of two or more readings on each sample, blank or standard; therefore, all the microcomputer has to do is to recognize the mean result and store it.

When used in conjunction with an autosampler, these instruments will usually provide an option to periodically zero the absorbance readings on blanks and reslope on standards. However, where there is appreciable base-line or sensitivity drift between standards, significant errors can result, particularly in samples immediately prior to recalibration (see figure 2[*a*]). A more accurate approach is to read blanks and standards as if they were samples then retrospectively apply base-line and sensitivity correction to sample data by time-based interpolation between blanks and standards (see figure 2[*b*]). No use is made of the instrument microprocessor's ability to auto-zero or reslope during the run since these functions are performed externally; however, curve correction is applied by the instrument during initial calibration, since there is no advantage in performing this externally. Final results are not calculated until the end of a run of samples, but this does not result in any inconvenience since the analyst is rarely in attendance during long automated sample runs.

### Suspended solids

This is a measurement of weight differences in dried filter papers before and after filtration of known volumes of samples. Raw data required comprise initial weight of filter (*W*1 g), volume of sample taken for filtration (*V* ml) and final weight of filter with solids (*W*2 g); calculation of suspended solids is given by $10^6 \times (W2-W1)/V$ mg/l.

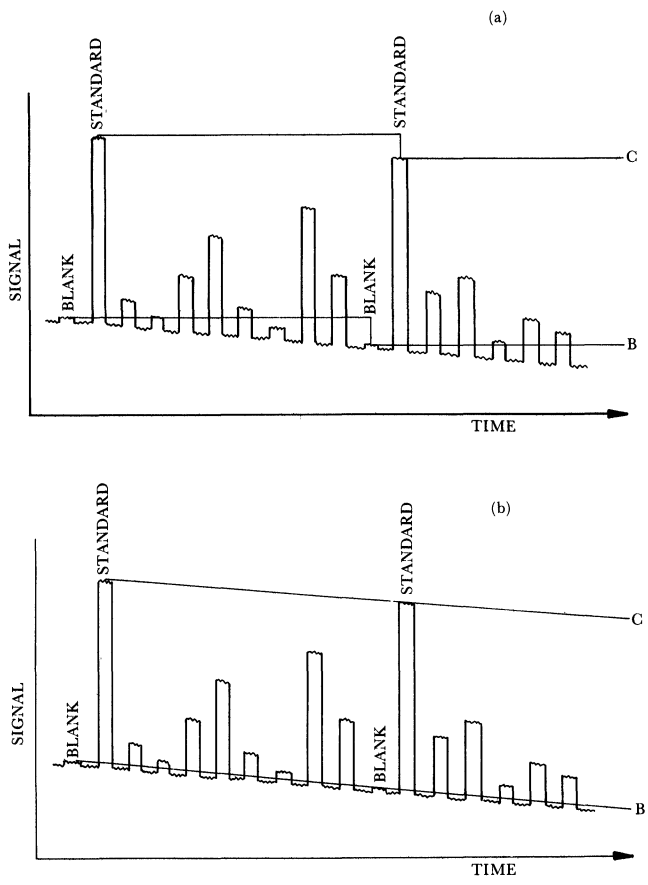

*Figure 2. Flame AAS traces indicating assignment of base-line (B) and calibration (C) by an instrument microprocessor (figure 2[a]) and an external microcomputer (figure 2[b]).*

The procedure adopted before the introduction of a microcomputer involved hand-written records of all raw data, followed by keyboard entry onto the laboratory computer for calculation of results. Occurrence of transcription errors was not uncommon, perhaps not surprisingly since five-place decimal weighings are recorded. Having purchased an electronic balance with RS232 output it required only a simple program on a Tandy Model 100 to record weighings and download calculated results to the laboratory computer.

The balance, a Mettler AE163, is operated by a footswitch which, when pressed, triggers an output to the computer, but only when the balance registers a stable reading. Integration time and stability level may be selected on the balance. The computer can be programmed to only accept readings within a certain range in order to avoid recording zero weights if the footswitch is operated accidentally between filter weighings. A fast routine for manually entering sample volumes is provided in which a default volume of 200 ml is used so that only volumes other than this value need to be entered by the analyst. Raw data may be displayed, edited or printed before serial transmission of final results to the laboratory computer. More than 15 000 suspended solids results per year are determined in this way. Additionally, total dissolved solids are processed using the same program.

### Biochemical oxygen demand

Measurement of dissolved oxygen (DO) in a sample before and after a five-day incubation period at 20 °C is the standard method for estimating biochemical oxygen demand (BOD). Raw data required for the test comprise initial DO (D1), sample dilution factor (d) and final DO (D2); calculation of mg/l BOD is given by d × (D2–D1) − (d–1) × B/d, where B is the measured BOD of the dilution water. It can be seen that DO readings and dilutions used in this calculation are analogous to weights and volumes used in the suspended solids calculation above. It therefore took little effort to adapt the suspended solids software for BOD determinations, with additional programming to allow for blank subtraction.

DO readings are taken from a Corning 155 digital pH/mV meter incorporating an RS232 output. Two EIL dissolved oxygen probes are alternately connected through a 'Y' switch so that one can be read whilst the other is equilibrating with fresh sample. Output is initiated at the meter which awaits a stable reading before transmission to the microprocessor. An audible signal is produced on signal transmission which leaves the analyst free to make up sample dilutions during stabilizing periods. Alternatively, an alarm sounds if the initial DO reading is too low so that the sample may be further diluted prior to incubation.

Owing to the length of time taken to complete BOD tests, i.e. five days, and the possibility of accidentally losing data from the RAM files of the microcomputer, tape securities of raw data files are kept until the final results are downloaded to the laboratory computer. As with suspended solids, determined raw data can be validated and printed before final results are transmitted to the laboratory computer. Approximately 10 000 BOD determinations per annum are carried out at Malvern.

### Continuous flow analysis

Many routine tests carried out at Malvern are performed using the technique of continuous flow analysis (CFA). Although there is a current trend in water authority laboratories towards automated discrete analysis, the Malvern Laboratory has such a large investment in CFA apparatus that its replacement cannot yet be justified on either cost or efficiency grounds. Raw data obtained on Technicon AAII CFA equipment for routine tests such as ammonia, TON, chloride, etc., are processed through the Series/1 computer via a tape logger and replay unit (see figure 1).

Certain other tests are run less frequently; these are more conveniently handled through an independent channel of processing. This alternative system uses a Model 12 microcomputer in conjunction with a solid-state, analogue-to-digital, data-logging device, the Grant Squirrel. The Squirrel stores up to 4000 readings on each of two channels, although single- or four-channel versions are also available. Recording intervals are selectable in 1s increments up to 100 min, but typically 2 to 5s intervals are appropriate for most CFA systems in order that sufficient readings are taken for peak definition.

Once the Squirrel-logged data have been transmitted to a microcomputer, a peak-picking routine is applied for which the computer requires the following information:

(1) Expected number of peaks.
(2) Approximate time per peak.
(3) Recording interval.
(4) Threshold value for first peak.
(5) Minimum value for assignment of peak maximum.

The last item is required so that small peaks are omitted from the peak maxima assignment routine. Consequently, long intervals in which there are no significant peaks place great reliance on the timing accuracy of the autosampler and the measured time per peak. In practice, a standard should be inserted at least every 10 samples to eliminate this problem and, at the same time, provide more reliable calibration.

### Management information

Timely management information is essential for the efficient and effective management of a laboratory. Key areas for which information must be collected are:

(1) *Levels of service:* this will include 'quality' characteristics such as time between receipt of sample and reporting of results, and analytical quality control (AQC) statistics.
(2) *Work-load:* this is quantitative information such as numbers of samples, determinations, standards etc., usually collected over several time periods, for example daily, weekly or monthly.
(3) *Efficiency:* this can be measured on several criteria, usually combining quantitative information with one or more of manpower, time, total laboratory cost etc. to produce unit cost figures.

The Series/1 computer automatically collects data and produces reports on (1) and (2) above, but manipulation of data to produce information on efficiency and unit costs is better achieved using a microcomputer. Bulk data are transferred from the Series/1 to a Tandy Model 12 microcomputer via 8 in diskettes and the appropriate additional information (manpower figures, hours worked, total costs etc.) added both manually and from data stored on a Model 100 microcomputer. The combined data are processed on the Model 12 in order to produce management information summary reports as required.

### Other applications

Microcomputers can often assist the analyst in a number of less obvious ways. For instance, there are always repetitive calculations to be performed which are more conveniently run on a microprocessor rather than a calculator. At Malvern, these include determinations of ionic balance, dry solids, organic matter, metals equivalents and chemical oxygen demand. Furthermore, the ability to use laboratory computers as word processors when not engaged in analytical work can be of enormous benefit. Any laboratory method can now be recalled from floppy disk so that alterations to text can be made and a revised version obtained immediately.

There is currently considerable interest in the use of microprocessor-controlled robot arms for sample preparation in laboratories. However, the inhomogeneous nature of many samples of interest to the water industry at present precludes the use of robots in areas of sample preparation where representative subsamples are required. For example, there is not yet available a robot arm capable of lifting and shaking a litre bottle of liquid containing suspended material and removing an appropriate volume subsample for the BOD test. It is likely that future advances in technology will overcome these difficulties, otherwise the quest for increased automation and efficiency may bring about changes in emphasis placed on such tests.

### Conclusions

This paper shows some applications of microcomputers in a water authority laboratory; these have demonstrated the following benefits:

(1) Increased reliability of data.
(2) Time savings.
(3) Increased efficiency.
(4) Enhanced flexibility.
(5) Improved management information.

However, the introduction of such rapidly developing technology in the laboratory must be done with flexibility and compatibility in mind, whilst maximizing the use of existing laboratory instruments.

### Acknowledgement

We wish to thank Mr V. W. Howells, Divisional Scientist, Lower Severn Division, S.T.W.A., for his helpful comments on the manuscript.

### NOTES FOR AUTHORS

*Journal of Automatic Chemistry incorporating Journal of Clinical Laboratory Automation* covers all aspects of automation and mechanization in analytical, clinical and industrial environments. The *Journal* publishes original research papers; short communications on innovations, techniques and instrumentation, or current research in progress; reports on recent commercial developments; and meeting reports, book reviews and information on forthcoming events. All research papers are refereed.

### Manuscripts

Two copies of articles should be submitted. All articles should be typed in double spacing with ample margins, on one side of the paper only. The following items should be sent: (1) a title-page including a brief and informative title, avoiding the word 'new' and its synonyms; a full list of authors with their affiliations and full addresses; (2) an abstract of about 250 words; (3) the main text; (4) appendices (if any); (5) references; (6) tables, each table on a separate sheet and accompanied by a caption; (7) illustrations (diagrams, drawings and photographs) numbered in a single sequence from 1 upwards and with the author's name on the back of every illustration; captions to illustrations should be typed on a separate sheet. Papers are accepted for publication on condition that they have been submitted only to this *Journal.*

### References

References should be indicated in the text by numbers following the author's name, i.e. Skeggs [6]. In the reference section they should be arranged thus:

to a journal

Manks, D. P., *Journal of Automatic Chemistry*, **3** (1981), 119.

to a book

Malmstadt, H. V., in *Topics in Automatic Chemistry*, Ed. Stockwell, P. B. and Foreman, J. K. (Horwood, Chichester, 1978), p. 68.

### Illustrations

Original copies of diagrams and drawings should be supplied, and should be drawn to be suitable for reduction to the page or column width of the *Journal*, i.e. to 85 mm or 179 mm, with special attention to lettering size. Photographs may be sent as glossy prints or as negatives.

### Proofs and offprints

The principal or corresponding author will be sent proofs for checking and will receive 50 offprints free of charge. Additional offprints may be ordered on a form which accompanies the proofs.

*Manuscripts should be sent to either Dr P. B. Stockwell or Ms M. R. Stewart, see inside front cover.*

